# HIV-1 Nef-mediated downregulation of CD155 results in viral restriction by KIR2DL5^+^ NK cells

**DOI:** 10.1371/journal.ppat.1010572

**Published:** 2022-06-24

**Authors:** Pia Fittje, Angelique Hœlzemer, Wilfredo F. Garcia-Beltran, Sarah Vollmers, Annika Niehrs, Kerri Hagemann, Glòria Martrus, Christian Körner, Frank Kirchhoff, Daniel Sauter, Marcus Altfeld

**Affiliations:** 1 Leibniz Institute of Virology (LIV), Hamburg, Germany; 2 First Department of Internal Medicine, Division of Infectious Diseases, University Medical Center Hamburg-Eppendorf, Hamburg, Germany; 3 German Center for Infection Research (DZIF), Partner Site Hamburg-Lübeck-Borstel-Riems, Hamburg, Germany; 4 Department of Pathology, Massachusetts General Hospital/Harvard Medical School, Boston, Massachusetts, United States of America; 5 Institute of Molecular Virology, Ulm University Medical Center, Ulm, Germany; 6 Institute for Medical Virology and Epidemiology of Viral Diseases, University Hospital Tübingen, Tübingen, Germany; University of Wisconsin, UNITED STATES

## Abstract

Antiviral NK cell activity is regulated through the interaction of activating and inhibitory NK cell receptors with their ligands on infected cells. HLA class I molecules serve as ligands for most killer cell immunoglobulin-like receptors (KIRs), but no HLA class I ligands for the inhibitory NK cell receptor KIR2DL5 have been identified to date. Using a NK cell receptor/ligand screening approach, we observed no strong binding of KIR2DL5 to HLA class I or class II molecules, but confirmed that KIR2DL5 binds to the poliovirus receptor (PVR, CD155). Functional studies using primary human NK cells revealed a significantly decreased degranulation of KIR2DL5^+^ NK cells in response to CD155-expressing target cells. We subsequently investigated the role of KIR2DL5/CD155 interactions in HIV-1 infection, and showed that multiple HIV-1 strains significantly decreased CD155 expression levels on HIV-1-infected primary human CD4^+^ T cells via a Nef-dependent mechanism. Co-culture of NK cells with HIV-1-infected CD4^+^ T cells revealed enhanced anti-viral activity of KIR2DL5^+^ NK cells against wild-type versus Nef-deficient viruses, indicating that HIV-1-mediated downregulation of CD155 renders infected cells more susceptible to recognition by KIR2DL5^+^ NK cells. These data show that CD155 suppresses the antiviral activity of KIR2DL5^+^ NK cells and is downmodulated by HIV-1 Nef protein as potential trade-off counteracting activating NK cell ligands, demonstrating the ability of NK cells to counteract immune escape mechanisms employed by HIV-1.

## Author summary

HIV infection remains a global health emergency that has caused around 36 million deaths. NK cells play an important role in the control of HIV-1 infections, and are able to detect and destroy infected cells using a large array of activating and inhibitory receptors, including KIRs. Here we demonstrate that CD155 serves as a functional interaction partner for the inhibitory NK cell receptor KIR2DL5, and that KIR2DL5^+^ NK cells are inhibited by CD155-expressing target cells. CD155 surface expression on HIV-1-infected CD4^+^ T cells was downregulated by the HIV-1 Nef protein, resulting in increased anti-viral activity of KIR2DL5^+^ NK cells through the loss of inhibitory signals. Taken together, these studies demonstrate functional consequences of the novel interaction between KIR2DL5 and CD155 for the antiviral activity of KIR2DL5^+^ NK cells during HIV-1 infection.

## Introduction

Natural killer (NK) cells are important antiviral effector cells of the innate immune system. NK cells can recognize virus-infected cells through activating receptors and the loss of engagement of inhibitory receptors [1], enabling both tolerance against self and effective immune responses against virus-infected and tumor cells [2]. One important NK cell receptor family is the group of killer cell immunoglobulin-like receptors (KIRs), which contains several structurally related activating and inhibitory receptors. To date, all described functional ligands for KIRs constitute HLA class I molecules, including KIR2DL1 and KIR2DL3 binding to HLA-C group 2 and 1, respectively, and KIR3DL1 binding to HLA-Bw4 molecules [3–5]. While for most KIRs a functional ligand has been defined, the inhibitory KIR2DL5 was long considered an “orphan” receptor [6]. Like other KIRs, KIR2DL5 is genetically polymorphic and due to a duplication of the gene in humans encoded by two different loci on chromosome 19 designated as *KIR2DL5A* and *KIR2DL5B*. However, surface expression has been mainly detected for molecules encoded by the *KIR2DL5A* alleles [6,7]. Recently, the poliovirus receptor (PVR/CD155) has been described as a binding partner for KIR2DL5 [8,9], potentially identifying a KIR not to interact with HLA class I molecules. Given the well-established functional interactions of CD155 with the activating NK cell receptors DNAM-1 [10] and CD96 [11] and the inhibitory NK cell receptor TIGIT [12], the newly described binding of KIR2DL5 to CD155 suggests a complex regulation of NK cell activity by CD155. However, the functional consequences of KIR2DL5-CD155 interactions for primary NK cells and their antiviral activity remained unknown.

Viruses have evolved multiple strategies to evade immune cell recognition, including mechanisms to reduce the surface expression of ligands for immune cells on infected cells. These processes include the downregulation of HLA class I molecules by the HIV-1 accessory proteins Nef and Vpu to evade CD8^+^ T cell recognition. While Nef is involved in the downregulation of HLA-A and -B, Vpu reduces the surface expression of HLA-C on HIV-1-infected cells [13–16]. However, downregulation of HLA class I molecules can result in enhanced “missing-self” recognition of infected cells by NK cells through the loss of inhibitory signals mediated by inhibitory NK cell receptors binding to HLA class I [17,18]. In addition, HIV-1 can evade NK cell recognition by decreasing surface expression-levels of ligands for activating NK cell receptors such as MICA and MICB [19,20] that serve as ligands for the C-type lectin receptor NKG2D [21]. Recent data have suggested that CD155 surface expression can also be downmodulated by HIV-1, involving the viral proteins Nef and/or Vpu, probably to evade DNAM-1-dependant NK cell recognition of infected cells [22–25]. However, modulation of CD155 surface expression by HIV-1 remains incompletely understood, as some studies also suggest an upregulation [26] or no modulation of CD155 expression [20,27]. As the recently described interaction between KIR2DL5 and CD155 indicates a more complex regulation of NK cell function through CD155, we investigated the functional consequences of HIV-1-mediated regulation of CD155 expression for KIR2DL5^+^ NK cells. We show that CD155 serves as an important functional ligand for the inhibitory NK cell receptor KIR2DL5 that can inhibit primary human KIR2DL5^+^ NK cell activity. HIV-1 strains decreased CD155 expression levels on HIV-1-infected CD4^+^ T cells through a Nef-dependent mechanism, potentially in an effort to evade DNAM-1-mediated recognition by NK cells. However, this resulted in better *in vitro* inhibition of replication of wild-type viral strains by KIR2DL5^+^ NK cells compared to ΔNef viruses. Taken together, this study provides new functional insights into the interaction between KIR2DL5 and CD155, and the consequences for antiviral activity of KIR2DL5^+^ NK cells during HIV-1 infection.

## Results

### KIR2DL5 represents an additional binding partner for CD155

**Fig 1:**
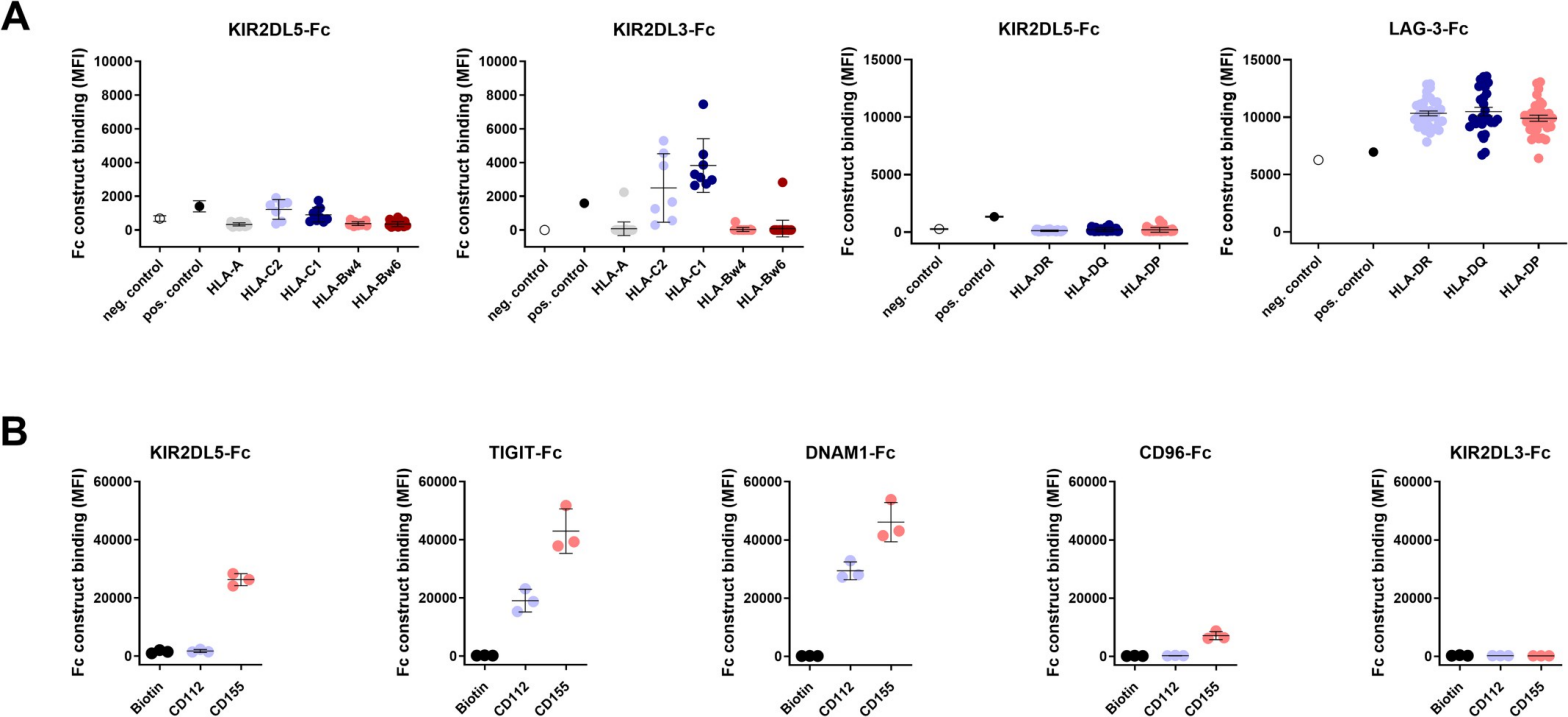
Binding of KIR-, TIGIT-, DNAM-1- and CD96-Fc constructs to HLA class I-, HLA class II- and nectin(-like)-coated beads. (A) KIR2DL5-Fc construct binding to HLA class I-coated and HLA class II-coated beads was measured by using a PE-labeled anti-IgG antibody and is shown as median fluorescence intensity (MFI). Each dot represents an individual HLA class I or class II allotype. Binding of KIR2DL5-Fc constructs was assessed in triplicates (*n* = 3). MFIs are shown as mean values for each HLA allotype. The 97 HLA class I allotypes as well as the 95 HLA class II allotypes were grouped according to the different subsets HLA-A, HLA-C1, HLA-C2, HLA-Bw4, HLA-Bw6 (HLA class I) and HLA-DR, HLA-DQ, HLA-DP (HLA class II). Negative control beads (white) were not coated with HLA antigen, positive control beads (black) were coated with purified human IgG. Black bars represent the mean of each HLA group and error bars show the standard deviation. (B) NK cell receptor Fc construct binding to CD112 (Nectin-2) and CD155 (PVR) was measured by flow cytometry. Binding of KIR2DL3, KIR2DL5, TIGIT, DNAM-1 and CD96 to biotin (neg. control), CD112 and CD155 was assessed as MFI in three independent experiments (*n* = 3). The mean values of the experiments are shown as black bars and standard deviations are depicted as error bars.

KIR2DL5 is an inhibitory NK cell receptor for which functional ligands are not well defined. To assess potential binding of KIR2DL5 to various HLA class I and HLA class II molecules, we performed a bead-based screening assay. NK cell receptor Fc fusion constructs, consisting of the extracellular domain of an NK cell receptor fused to an IgG1 Fc domain, were used to stain HLA-coated beads using an array of color-coded beads coated with 97 different HLA class I and 95 HLA class II molecules [28]. The NK cell receptor KIR2DL3 that recognizes HLA-C group 1 (HLA-C1) molecules [4] was used as a positive control for the HLA class I binding assay, and the lymphocyte activation gene 3 (LAG-3) protein, which is a high affinity ligand for HLA class II molecules [29], served as a positive control for the HLA class II screen (Fig 1A). While the KIR2DL3-Fc construct did interact with HLA-C1, KIR2DL5-Fc showed no strong binding to any of the investigated HLA class I molecules. Furthermore, LAG-3 was interacting with all HLA class II molecules, while KIR2DL5 did not bind to any of the HLA class II-coated beads (Fig 1A).

Nectin and nectin-like molecules play an important regulatory role for NK cell function by interacting with several NK cell receptors including TIGIT, DNAM-1 and CD96 [30]. To investigate whether members of the KIR family do also bind to nectin(-like) molecules, binding of TIGIT-, DNAM-1-, CD96- and KIR- (KIR2DL1, KIR2DL3, KIR2DL4, KIR2DL5 and KIR3DL1) Fc constructs to CD112 (Nectin-2)- and CD155 (PVR)-coated beads was determined. Beads coated with biotin served as negative control. These experiments confirmed previously described binding of nectin(-like) molecules to TIGIT, DNAM-1 and CD96 [10–12]. TIGIT and DNAM-1 exhibited the strongest interaction with CD155 and also bound to CD112 with lower affinity. CD96 is an intermediate affinity ligand for CD155 [30], and we also observed CD96-Fc construct binding to CD155 and no binding to CD112. Most investigated KIR-Fc constructs (KIR2DL1, KIR2DL3, KIR2DL4 and KIR3DL1) did not show any interaction with biotin-, CD112- or CD155-coated beads. However, KIR2DL5-Fc constructs exhibited binding to CD155, showing a slightly lower binding signal compared to DNAM-1 and TIGIT (Figs 1B and S1). Taken together, these data demonstrate that KIR2DL5, unlike other KIRs, does not strongly interact with HLA class I molecules, but binds to CD155.

### CD155 serves as a functional ligand for KIR2DL5

**Fig 2:**
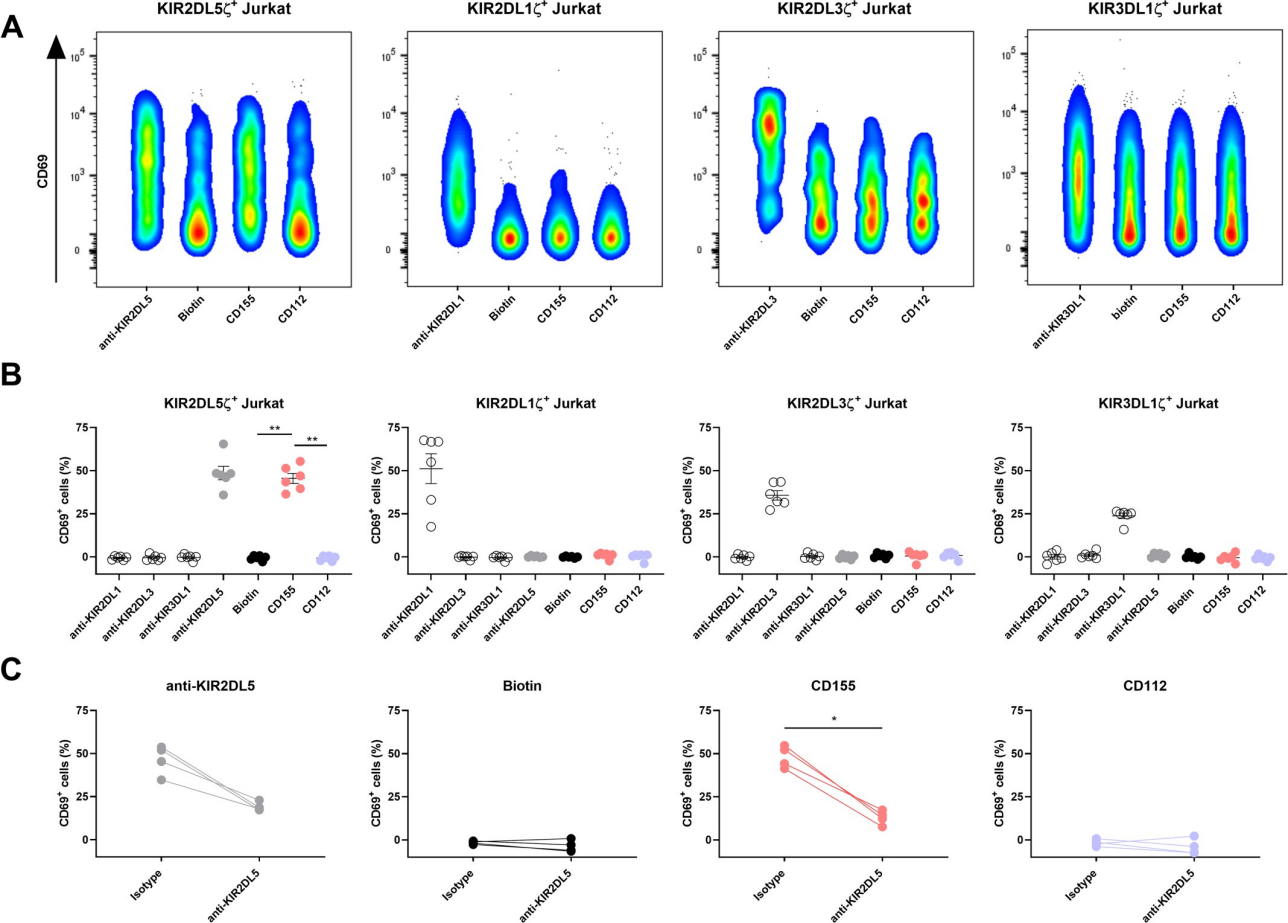
KIR2DL5ζ-expressing reporter cells are activated by interacting with CD155. (A) Reporter cell activity was determined by the upregulation of CD69 on the surface of KIR2DL1ζ, KIR2DL3ζ, KIR2DL5ζ and KIR3DL1ζ reporter cells during co-incubation with beads coated with anti-KIR2DL1, anti-KIR2DL3, anti-KIR2DL5 or anti-KIR3DL1, respectively, (pos. controls), biotin (neg. control), CD112 or CD155. Plots represent one out of six experiments and only the respective anti-KIR positive control is shown. (B) Percentage of CD69^+^ reporter cells after co-incubation with the indicated antibodies, biotin and nectin(-like) molecules. Plots show the results of six independent experiments (*n* = 6) with the mean value for each condition (black bar) and error bars depicting the standard error of the mean. Background activation (no target control) was subtracted from all samples. Mann-Whitney test was used to statistically analyze the difference in CD69 expression of CD155-, CD112- and biotin-stimulated cells (p = 0.0022). (C) Reporter cell activity of KIR2DL5ζ cells was assessed after incubating cells with purified anti-KIR2DL5 or mouse IgG isotype control antibody prior to co-incubation with anti-KIR2DL5-, biotin-, CD155- and CD112-coated beads. The percentage of CD69^+^ cells following incubation without beads (no target control) was subtracted from all samples. Reporter cell activity was determined in four independent experiments (*n* = 4). Lines between dots connect the matching samples incubated with the IgG isotype antibody or with purified anti-KIR2DL5. Mann-Whitney test was used to calculate statistical significance of differences in reporter cell activation through co-incubation with CD155-coated beads with and without KIR2DL5 blocking antibody (p = 0.0286).

While binding of KIR2DL5 to CD155 has previously been shown [8,9], we were interested in investigating the functional consequences of this interaction for primary NK cells. We therefore generated a KIR2DL5-expressing reporter cell line to further validate the interaction on a cellular basis by fusing the extracellular domain of KIR2DL5 to the intracellular part of the CD3ζ chain and stably expressing the chimeric construct within Jurkat cells. KIR2DL5 receptor binding to respective ligands can be determined based on CD69-upregulation on the surface of the KIR2DL5ζ reporter cells. Reporter cell lines transfected with KIR2DL1ζ, KIR2DL3ζ and KIR3DL1ζ were used as controls. KIRζ reporter cells were co-incubated with CD155-coated beads as well as with anti-KIR- (pos. control), biotin- (neg. control) and CD112-coated beads. All tested reporter cell lines (KIR2DL1ζ, KIR2DL3ζ, KIR2DL5ζ and KIR3DL1ζ) upregulated CD69 expression upon co-incubation with their respective anti-KIR antibody (pos. control beads; compared to unstimulated controls) and showed no functional response to biotin-coated beads (neg. control beads) (Fig 2A and 2B). Only KIR2DL5ζ reporter cells showed a significant upregulation of CD69 expression upon incubation with CD155-coated beads compared to the co-incubation with negative control beads (biotin) or beads coated with CD112 (p < 0.01) (Figs 2A, 2B and S2). Blocking experiments using antibodies directed against KIR2DL5 or a respective isotype control were performed. The isotype antibody did not influence reporter cell activation following co-incubation with anti-KIR2DL5- and CD155-coated beads, whereas the anti-KIR2DL5 antibody significantly abrogated CD69 upregulation on KIR2DL5ζ reporter cells (p < 0.05) (Fig 2C). Taken together, these data demonstrate that the interaction between KIR2DL5 and CD155 results in the functional activation of KIR2DL5ζ reporter cells.

### KIR2DL5^+^ primary human NK cells are inhibited by CD155-expressing target cells

**Fig 3:**
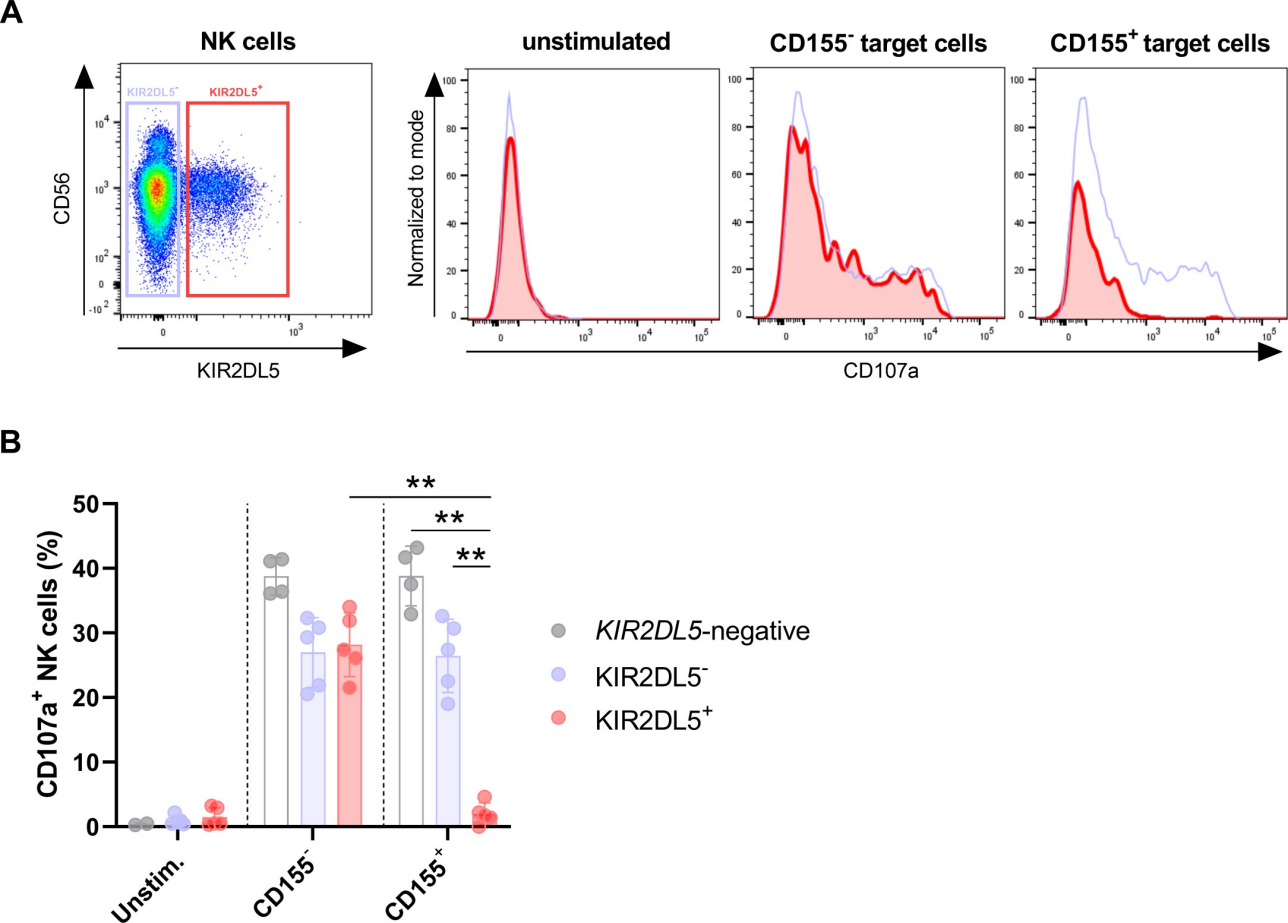
Primary human KIR2DL5^+^ NK cells are inhibited by CD155-expressing target cells. (A) Degranulation of KIR2DL5^+^ (red) and KIR2DL5^-^ (blue) NK cells was defined as percentage of CD107a^+^ NK cells after co-incubation with CD155^-^ 721.221 target cells, CD155^+^ 721.221 target cells or left unstimulated. Plots show one representative experiment out of five independent experiments. (B) Percentage of CD107a-positive NK cells after co-incubation with target cells is shown as indicated before. Bars indicate mean values with standard deviations of KIR2DL5^+^ donors (*n* = 5 from 5 donors) and *KIR2DL5*-negative donors (*n* = 4 from 2 donors). Mann-Whitney test was used to calculate statistical significance of differences in CD107a levels (KIR2DL5^-^ versus KIR2DL5^+^ NK cells p = 0.008; *KIR2DL5*-negative versus KIR2DL5^+^ NK cells p = 0.016; KIR2DL5^+^ NK cells co-cultured with CD155^+^ or CD155^-^ target cells p = 0.008).

To determine the consequences of interactions between KIR2DL5 and CD155 for primary human KIR2DL5^+^ NK cell function, NK cell degranulation upon co-incubation with 721.221 cells, which do not express CD155 at the cell surface, and transduced 721.221 cells overexpressing CD155 (S3 Fig), was assessed. NK cells of a KIR2DL5A*001-positive donor and a donor completely lacking KIR2DL5 genetically were co-cultured with CD155^+^ or CD155^-^ target cells, and CD107a expression levels on KIR2DL5^-^ and KIR2DL5^+^ NK cells served as readout for NK cell degranulation in response to the different target cells (Fig 3A). All NK cell populations (KIR2DL5^-^, KIR2DL5^+^ and NK cells from *KIR2DL5*-negative donors) showed increased CD107a expression after co-incubation with CD155^-^ 721.221 cells (Fig 3B). When co-incubating NK cells with CD155^+^ 721.221 cells, KIR2DL5^+^ NK cells expressed significantly lower CD107a levels compared to KIR2DL5^-^ NK cells (p < 0.01) and NK cells of *KIR2DL5*-negative donors (p < 0.02), and compared to KIR2DL5^+^ NK cells co-cultured with CD155^-^ target cells (p < 0.01) (Fig 3A and 3B). Taken together, these data show that interactions between CD155 and KIR2DL5 result in functional inhibition of primary human KIR2DL5^+^ NK cells.

### HIV-1 mediates Nef-dependent downregulation of CD155 on HIV-1-infected CD4^+^ T cells

**Fig 4:**
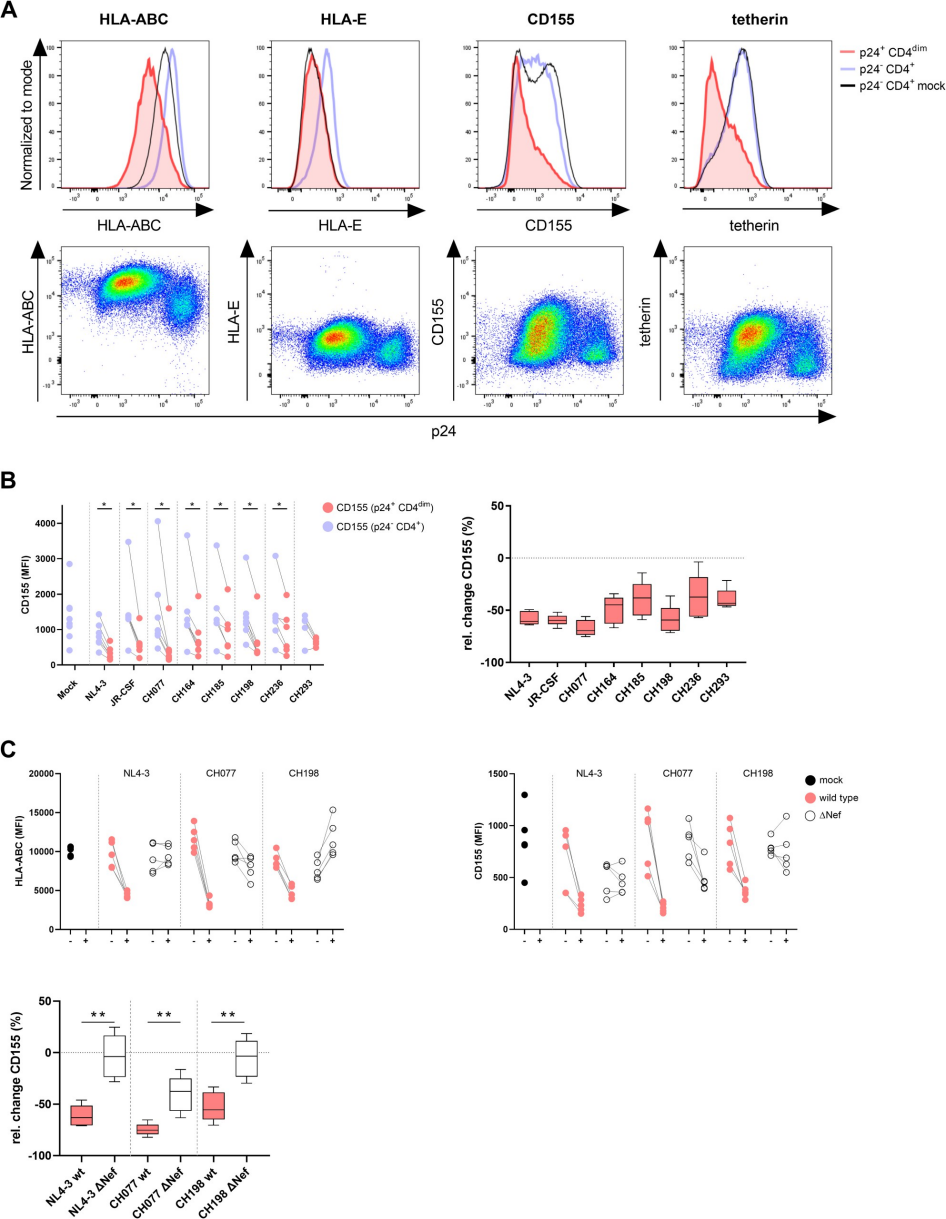
HIV-1 mediates downregulation of CD155 on infected CD4^+^ T cells in a Nef-dependent manner. (A) Expression levels of HLA-ABC, HLA-E, CD155 and tetherin were compared between HIV-1-infected (CH077) and uninfected CD4^+^ T cells. HIV-1-infected cells were determined by gating on p24^+^ CD4^dim^ cells and uninfected cells were defined as p24^-^ and CD4^+^. Histograms show surface expression of the indicated molecules on HIV-1-infected (red), uninfected (blue) and mock infected (black) CD4^+^ T cells. Dot plots display surface expression of HLA-ABC, HLA-E, CD155 and tetherin on p24^-^ and p24^+^ CD3^+^ T cells. (B) Modulation of CD155 expression on HIV-1-infected CD4^+^ T cells was assessed by using different HIV-1 cell line-adapted (NL4-3, JR-CSF) and primary strains (CH077, CH164, CH185, CH198, CH236, CH293). Mock-infected cells served as a control. The left graph shows CD155 expression on uninfected (blue) compared to infected (red) cells depicted as MFI. Each dot represents the result of one independent experiment. The right graph shows the median relative change (%) of CD155 expression mediated by the different HIV-1 strains, which was calculated as (MFI infected–MFI uninfected)/MFI uninfected × 100. HIV-1 strains (NL4-3, JR-CSF, CH077, CH164, CH185, CH198 and CH236) showed a significant downregulation of CD155 on infected cells (NL4-3, CH077, CH164, CH198 p = 0.016; JR-CSF, CH185, CH236 p = 0.03), calculated by performing the Wilcoxon matched-pairs signed rank test. Samples <150 cells were excluded, resulting in varying numbers of donors per condition. (C) HIV-1 wild type (wt) strains that showed the strongest CD155 downregulation (NL4-3, CH077, CH198) were compared to ΔNef mutant viruses and analyzed for their ability to downmodulate HLA-ABC and CD155. Expression levels depicted as MFI are shown for mock (black), wt virus (red) and ΔNef virus (white) infected CD4^+^ T cells (n = 5). The median relative change of CD155 expression is shown for wt (red) and ΔNef mutant viruses (white). Mann-Whitney test was used to calculate statistical significance of differences between the downregulation of CD155 by HIV-1 wt and ΔNef mutant viruses (p = 0.008).

HIV-1 has developed several mechanisms to evade innate and adaptive immune responses by modulating the expression of cell surface molecules such as HLA class I [14,15,31]. Previous studies have suggested that HIV-1 can also modulate protein expression levels of CD155 on the surface of infected cells. However, CD155 expression in HIV-1 infection is under debate, as some studies described a downregulation [22–25] whereas others reported no effect or an upregulation [20,26,27]. While most previous studies used tumor-transformed cell lines as infection models and/or pseudotyped HIV-1 particles, we decided to determine how genuine HIV-1 strains, including primary transmitted-founder HIV-1 infectious molecular clones (IMCs), impact CD155 expression on primary CD4^+^ T cells. Freshly isolated human CD4^+^ T cells were infected with cell line-adapted (NL4-3 and JR-CSF) or primary (CH077, CH164, CH185, CH198, CH236, CH293) HIV-1 strains and median fluorescence intensity (MFI) levels of HLA class I (HLA-I), HLA-E, tetherin and CD155 were compared between HIV-1-infected p24 Gag^+^ CD4^dim^ cells and uninfected p24 Gag^-^ CD4^+^ cells within the same experimental tube (Figs 4A and S4). In line with previous studies [31–33], CD4^+^ T cells infected with different HIV-1 strains exhibited lower expression levels of HLA-I, HLA-E and tetherin than uninfected cells, indicating downregulation of these molecules by HIV-1 (exemplarily shown for the HIV-1 strain CH077 in Fig 4A). Similarly, the expression levels of CD155 were significantly decreased on HIV-1-infected cells compared to uninfected CD4^+^ T cells, and this downregulation was observed for all investigated laboratory-adapted and primary HIV-1 strains (Fig 4B). CD155 levels on uninfected bystander cells were similar to those on mock-infected CD4^+^ T cells of the same donor, which were treated the same but without adding virus (Fig 4A and 4B). CD155 levels depicted as relative change for the different HIV-1 strains are shown in Fig 4B and revealed the strongest downregulation of CD155 on CD4^+^ T cells infected with CH077, CH198 and NL4-3. Taken together, laboratory-adapted and primary HIV-1 strains downmodulate CD155 from the surface of primary human HIV-1-infected CD4^+^ T cells.

The HIV-1 accessory protein Nef is known to mediate downregulation of HLA-I cell surface expression [31], and was previously also suggested to be involved in CD155 modulation [22,24]. To investigate whether Nef contributes to the modulation of CD155 by CH077, CH198 and NL4-3, which showed the strongest downregulation of CD155, we used the respective wild type (wt) and Nef-defective mutant (ΔNef) viruses to infect CD4^+^ T cells. As Nef also targets CD4 surface expression, ΔNef-infected CD4^+^ T cells were defined as p24 Gag^+^ tetherin^-^ CD4^+^ T cells. All three tested HIV-1 wt strains induced downregulation of HLA-ABC on the surface of infected CD4^+^ T cells, which was not observed or less pronounced when infecting cells with the respective ΔNef mutant viruses (Fig 4C). While the wild type viruses also downmodulated CD155 as described above, the ΔNef mutants did not downregulate CD155 expression to the same extent (p < 0.01) (Fig 4C). These data demonstrate that the accessory HIV-1 protein Nef is involved in the downregulation of CD155 from the surface of infected primary human CD4^+^ T cells.

### Reduced *in vitro* inhibition of HIV-1 replication by KIR2DL5^+^ NK cells against Nef-deficient strains

**Fig 5:**
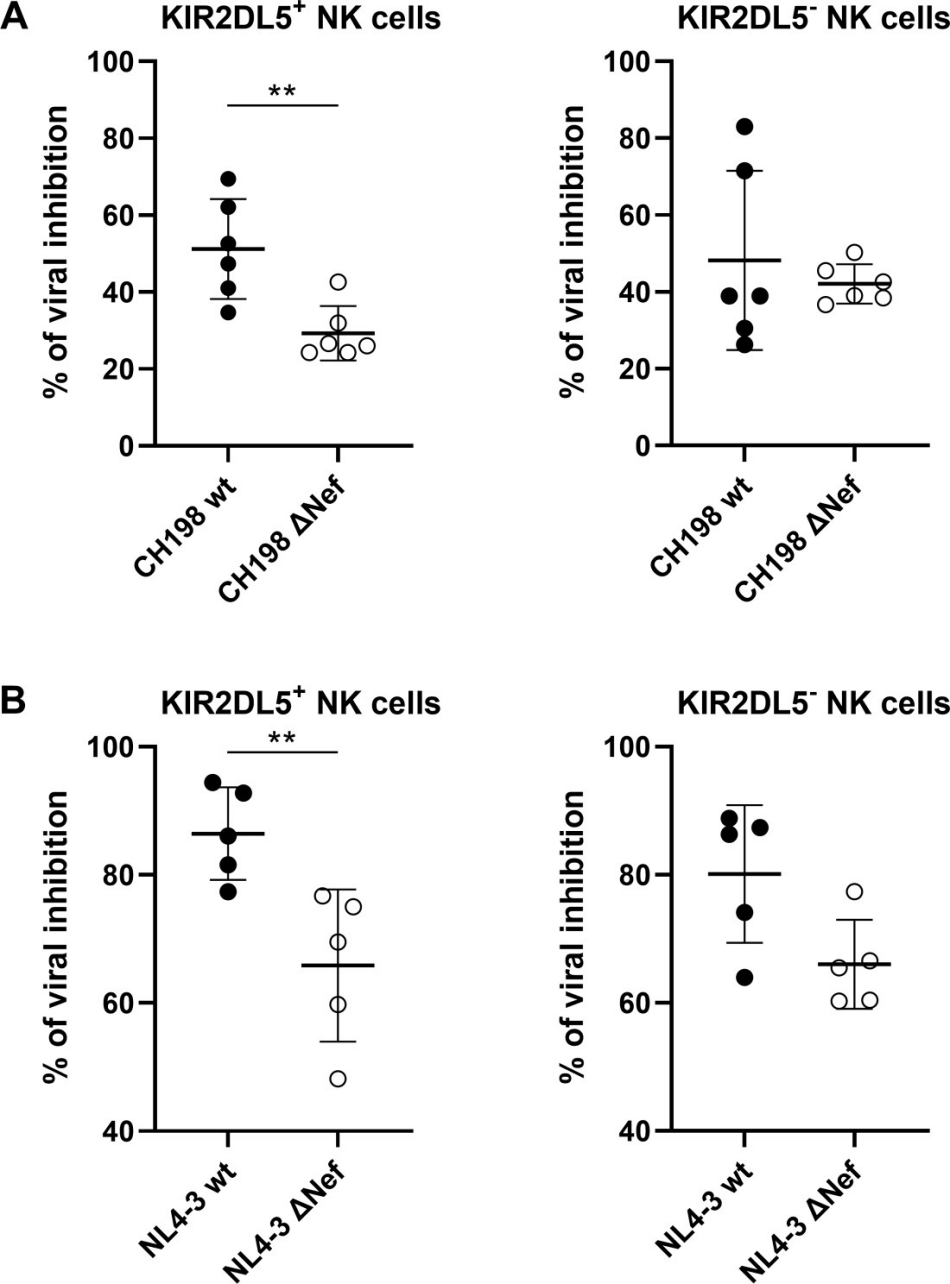
Inhibition of HIV-1 replication by KIR2DL5^+^ and KIR2DL5^-^ NK cells. Anti-viral activity of KIR2DL5^+^ and KIR2DL5^-^ NK cells was evaluated by co-culture of KIR2DL5^+^ and KIR2DL5^-^ NK cell clones with HIV-1 CH198 (A) and NL4-3 (B) wt and ΔNef virus infected autologous CD4^+^ T cells. Graphs show the percentage of viral inhibition based on the percentage of p24^+^ CD4^+^ T cells in co-culture with NK cells compared to the CD4^+^ T cell only control where no NK cells were added. Viral inhibition was calculated as 100*(1-(percentage of p24^+^ CD4^+^ T cells with NK cells / percentage of p24^+^ CD4^+^ T cells without NK cells)). Mann-Whitney test was used to calculate statistical significance of differences in viral inhibition between wt and ΔNef viruses (CH198: p = 0.009; NL4-3: p = 0.008). Data were obtained by using six (for CH198 viral inhibition) and five (for NL4-3 viral inhibition) KIR2DL5^+^ and six/five KIR2DL5^-^ NK cell clones from two donors (CH198: *n* = 6; NL4-3: *n* = 5) and results are shown as mean values with SD.

To evaluate the consequences of CD155-downregulation for the antiviral capacity of KIR2DL5^+^ NK cells, we co-incubated HIV-1 CH198 and NL4-3-infected (wt and ΔNef) CD4^+^ T cells with autologous KIR2DL5^+^ or KIR2DL5^-^ NK cell clones. While KIR2DL5^+^ and KIR2DL5^-^ NK cell clones differed significantly in the expression of KIR2DL5 (KIR2DL5 median fluorescence intensity (MFI) for KIR2DL5^+^ NK cell clones: 400; KIR2DL5 MFI for KIR2DL5^-^ NK cell clones: 8; p < 0.0001), expression of TIGIT, DNAM-1 and CD96 was similar between the clones (p > 0.1). After a co-incubation of 7 days, viral inhibition was assessed by quantifying the percentage of p24^+^ cells. KIR2DL5^+^ NK cells exhibited a significantly higher inhibition of viral replication of CH198 and NL4-3 wt viruses compared to the respective ΔNef viruses (p < 0.01), while only a small effect (NL4-3) or no effect (CH198) was observed for KIR2DL5^-^ NK cells (Fig 5A and 5B). These data indicate that the ability of KIR2DL5^+^ NK cells to inhibit HIV-1 replication *in vitro* is significantly affected by Nef-mediated regulation of CD155, while the ability of KIR2DL5^-^ NK cells is not. This was further supported by the observation that inhibition of replication of ΔNef CH198 (lacking the ability to downregulate CD155) was significantly lower by KIR2DL5^+^ NK cells compared to KIR2DL5^-^ NK cells (p < 0.02) (Fig 5A). Inhibition of replication of ΔNef NL4-3 by NK cells was more variable (Fig 5B). Taken together, these data demonstrate a significant impact of changes in CD155 expression observed between wild type and ΔNef strains for the ability of KIR2DL5^+^ NK cells to inhibit viral replication *in vitro*.

## Discussion

The different members of the KIR family contribute to the tight regulation of NK cell function by mediating inhibitory or activating signals through interactions with HLA class I. The inhibitory NK cell receptor KIR2DL5 is the most recently identified human KIR for which a ligand was not identified for almost two decades [6,34]. Due to the fact that ligands remained unknown, the functional relevance of KIR2DL5-expressing NK cells in human diseases is poorly understood. Recently, the nectin-like molecule CD155 was reported to interact with KIR2DL5 [8,9], making KIR2DL5 the first KIR that binds to other proteins than HLA class I molecules. Here, we demonstrate that CD155 serves as a functional ligand of KIR2DL5, mediating inhibitory signals resulting in decreased activity of primary KIR2DL5^+^ NK cells against CD155-expressing target cells. Furthermore, we show that the viral protein Nef decreases CD155 expression levels on HIV-1-infected primary human CD4^+^ T cells, leading to better inhibition of the *in vitro* replication of wild type HIV-1 strains compared to Nef-deficient strains by KIR2DL5^+^ NK cells. Taken together, these data demonstrate that KIR2DL5 is an important binding partner of CD155, modulating KIR2DL5^+^ NK cell-mediated immune responses against HIV-1-infected target cells.

The family of nectin and nectin-like molecules has been identified as a group of ligands for inhibitory and activating NK cell receptors, thereby contributing to the regulation of NK cell function [30]. CD155 is a member of the immunoglobulin superfamily and has central roles in cell adhesion and immune responses [30]. While the binding of CD155 to the activating NK cell receptors DNAM-1 [10] and CD96 [11] as well as to the inhibitory receptor TIGIT [12] is well established, the here described functional interaction between CD155 and the inhibitory NK cell receptor KIR2DL5 adds another axis to the complex regulation of NK cells by CD155. KIRs are known to interact with several of the highly polymorphic HLA class I molecules on the surface of normal cells, keeping NK cell effector functions tightly regulated. In contrast to other KIRs, including the structural closely related KIR2DL4 [6,35], KIR2DL5 is the first KIR for which no strong binding to any of the tested HLA molecules has been observed, indicating that HLA class I and class II might not represent the main binding partners for KIR2DL5. KIR2DL5 genes are present in all human populations with frequencies ranging between 26% and 86%, and are expressed on up to 10% of total NK cells in healthy individuals encoding for *KIR2DL5* [6]. Like other KIRs, KIR2DL5 is genetically polymorphic, and due to a duplication of the gene in humans, encoded by two gene loci (*KIR2DL5A* and *KIR2DL5B*). The fact that only a minority of allotypes, mainly KIR2DL5A allotypes and most prevalent KIR2DL5A*001, have been shown to be expressed on the cell surface [6,7] may explain the interaction of a receptor encoded by a polymorphic gene with a conserved ligand such as CD155. However, binding of other KIRs to HLA class I can be modulated by specific peptides loaded to HLA class I [36–39], and it is therefore possible that KIR2DL5 might bind to HLA molecules under specific conditions. Previously, KIR2DL5 was suggested to mediate inhibitory signals [7,8,40]; however, due to the lack of a well-defined cellular ligand the functional role of KIR2DL5 in the settings of infection or inflammation remained poorly understood. Here, we show inhibition of primary KIR2DL5^+^ NK cells mediated through the CD155-KIR2DL5 axis using KIR2DL5^+^ NK cells isolated from donors encoding for *KIR2DL5A*. The establishment of KIR2DL5 as an inhibitory receptor binding CD155, although to a lesser extent than DNAM-1 and TIGIT, emphasizes the complex regulation of NK cell function by nectin and nectin-like molecules during health and disease.

Many viruses avoid recognition by immune cells through the modulation of surface expression of activating ligands. HIV-1 evades NK cell- and T cell-mediated immune responses through several mechanisms, including the downregulation of HLA class I molecules and stress-induced ligands on the surface of infected cells [13,14,16,19,20]. Here, we show that HIV-1 strains are also capable of downregulating CD155 on the surface of infected CD4^+^ T cells, as previously reported [22–25]. In addition to the shown effects of Nef on CD155 expression, other studies have suggested an impact of Vpu on the downregulation of CD155 [22–24]. However, a Vpu-mediated regulation of CD155 remains controversial, as some studies described an involvement of Vpu [22,23] and some did not [27]. Furthermore, it has been shown that while Nef reliably downregulates CD155, the impact of Vpu varied between different HIV-1 strains [24]. CD155 serves as a binding partner of the activating NK cell receptor DNAM-1 [10]. Thus, downregulation of CD155 by HIV-1 has probably evolved in an effort to avoid DNAM-1-mediated NK cell recognition. The newly described interaction between the inhibitory KIR2DL5 and CD155 therefore has functional consequences for KIR2DL5^+^ NK cell responses against HIV-1. Due to the Nef-dependent downregulation of CD155 by HIV-1, infected cells became more vulnerable to recognition by KIR2DL5^+^ NK cells, which resulted in enhanced inhibition of replication of HIV-1 wt strains compared to HIV-1 ΔNef strains by KIR2DL5^+^ NK cells. Although it has been described that Nef-defective HIV-1 strains might have an overall lower replication capacity [41–45], KIR2DL5^+^ NK cells showed a significantly decreased inhibition of replication of Nef-deficient CH198 IMCs compared to KIR2DL5^-^ NK cells, indicating a KIR2DL5-dependent effect. Individuals encoding for the expressed *KIR2DL5A* gene might therefore have an advantage in controlling HIV-1 replication. A recent study revealed an association between KIR2DL5 and reduced mother-to-child transmission of HIV-1 in infants born by HIV-1-infected mothers [46], supporting a protective role for KIR2DL5. Given the limited expression of KIR2DL5 on the cell surface, future studies need to discriminate between *KIR2DL5A* and *KIR2DL5B* genotypes when studying implications of KIR2DL5 expression on transmission and disease outcomes, and investigate this in the context of TIGIT, as it has been reported that TIGIT expression is upregulated on NK cells during HIV-1-infection [47,48]. Furthermore, KIR2DL5 has been suggested to be beneficial for the outcome of several other infectious diseases, and might also play a role in the outcome of cancer, as CD155 is known to be overexpressed in tumors [49–53].

In this study, we provide novel insights into the regulation of KIR2DL5^+^ NK cell function by interactions with the newly described ligand CD155, revealing a novel checkpoint inhibitor target for immunotherapeutic approaches. Furthermore, we demonstrate that HIV-1-mediated downregulation of CD155 can result in enhanced *in vitro* inhibition of HIV-1 replication by KIR2DL5^+^ NK cells. While the inhibitory receptor TIGIT, which is expressed on T and NK cells and also binds to CD155, is already investigated as a target in anti-tumor [54] and anti-HIV-1 [55] therapies, the inhibitory interactions between CD155 and KIR2DL5 might represent a second axis that can be targeted by future therapeutic approaches.

## Materials and methods

### Ethics statement

Peripheral blood samples were obtained from healthy blood donors recruited at the University Medical Center Hamburg-Eppendorf, Hamburg, Germany. These donors provided written informed consent and studies were approved by the ethical committee of the Ärztekammer Hamburg (PV4780). All participants were adults.

### Primary cells and cell lines

Primary human peripheral blood mononuclear cells (PBMCs) were isolated from whole blood donations from healthy donors by performing density-gradient centrifugation. Isolated PBMCs were washed and cultured in complete R10 medium (RPMI-1640 medium (Sigma) supplemented with 10% fetal bovine serum (FBS, Sigma)). PBMCs were directly used for experiments or cell populations such as NK cells and CD4^+^ T cells were isolated as indicated in detail below. Enriched NK cells were cultured overnight in complete medium supplemented with 1 ng/ml IL-15 before they were used in functional assays. HEK293T cells (American Type Culture Collection (ATCC), Cat#CRL-11268) were cultured in Dulbecco’s Modified Eagle Medium (DMEM (Life Technologies)) supplemented with 10% FBS (D10). Jurkat E6.1 (ATCC, Cat#TIB-152), B-LCL 721.221 (RRID: CVCL_6263) [56] and RPMI-8866 (RRID:CVCL_1668) cells were maintained in complete R10 medium. Jurkat reporter cells are derived from the Jurkat clone E6.1 and lack the expression of β_2_-microglobulin (β_2_m-KO) [57]. Cells were engineered to express KIRζ chimeric constructs as previously described [57]. The KIR2DL1ζ, KIR2DL3ζ and KIR3DL1ζ cells were generated previously [36,57]. KIR2DL5ζ constructs were generated by fusing the extracellular and transmembrane domain of KIR2DL5 to the intracellular CD3ζ chain. Constructs for Jurkat reporter cells and constructs for generating CD155-expressing 721.221 cells were obtained from GeneArt GeneSynthesis (Thermo Fisher) and cloned into a lentiviral transfer vector encoding for a puromycin resistance. To generate Jurkat reporter cells and 721.221 cells expressing the gene of interest, cells were lentivirally transduced. To this end, HEK293T cells were transfected with a VSV-G envelope vector (pHEF-VSVG; NIH AIDS Reagent Program), an HIV-1 Gag-Pol packaging vector (psPAX2; NIH AIDS Reagent Program) and the transfer vector (p SFFV-IRES-Puro (pSIP)-ZsGreen) carrying the gene of interest by using Lipofectamine 3000 (Life Technologies). Lentiviral supernatant was harvested after 48 hours and used to transduce β_2_m-KO Jurkat cells and 721.221 cells. After 3 days, cells were selected in 1 μg/ml puromycin (Sigma-Aldrich). Cells were cultured in complete R10 medium and maintained with 1 μg/ml puromycin.

### Recombinant human KIR-Fc construct binding to ligand-coated beads

Streptavidin Dynabeads (Thermo Fisher Scientific) were coated with either 200 pmol biotinylated protein or 10 μg biotinylated IgG/mg Dynabeads. Ligand-screening was performed by coating with biotinylated CD155 (PVR) (AcroBiosystems), CD112 (Nectin-2) (BPSBiosciences) or biotin as a negative control and anti-KIR2DL5 as a positive control. To screen for interactions with HLA class I and class II molecules, the LABScreen Single Antigen Class I and II kits (OneLambda) were used. Negative control beads were not coated with HLA antigens and positive control beads were coated with purified human IgG. Recombinant human Fc constructs (KIR2DL1-Fc, KIR2DL3-Fc, KIR2DL4-Fc, KIR2DL5-Fc, KIR3DL1-Fc, CD96-Fc, DNAM-1-Fc, LAG-3-Fc, PVR(CD155)-Fc, TIGIT-Fc) (R&D Systems) were diluted to 250 μg/ml in PBS and co-incubated with coated beads for 15 min at 4°C at a final concentration of 25 μg/ml. Samples were washed and bead-bound Fc constructs were detected by a staining with F(ab)2 goat-anti-human IgG PE secondary antibody (Life Technologies) for 15 min at 4°C. Interactions between Fc construct and peptide-coated beads were either quantified by flow cytometry (LSR Fortessa (BD Biosciences)) or by using the Luminex xMAP technology on a Bio-Plex 200 (Bio-Rad Laboratories).

### KIRζ reporter cell assay

Streptavidin Dynabeads were coated with biotinylated PVR (CD155), Nectin-2 (CD112), biotin, anti-KIR2DL1, anti-KIR2DL3, anti-KIR2DL5 and anti-KIR3DL1 as described before. 2.5 x 10^4^ cells of each reporter cell line were seeded into a well of a tissue culture-treated 96-well plate and co-incubated with 10 μl of the protein-coated beads for 5 h at 37°C/5% CO_2_ in a final volume of 200 μl. For blocking experiments, prior to co-incubation with beads, KIR2DL5^+^ JRC were blocked for 30 min with 30 μg/ml purified anti-KIR2DL5 or 30 μg/ml purified IgG1 isotype control antibody. Blocking antibodies remained in the wells during the whole assay. After co-incubation, cells were washed with PBS and stained with the viability dye LIVE/DEAD Fixable Near-IR (Life Technologies), anti-CD3-BUV395 (clone UCHT1, BD Biosciences), anti-CD69-BV421 (clone FN50, Biolegend) and the appropriate KIR antibody conjugated to PE (anti-KIR2DL1-PE (clone REA284), anti-KIR2DL3-PE (clone REA147), anti-KIR2DL5-PE (clone UP-R1), anti-KIR3DL1-PE (clone DX9) (Miltenyi). Cells were fixed in CellFix (BD Biosciences) and CD69 expression as a readout for KIR crosslinking was analyzed by flow cytometry.

### NK cell degranulation assay

NK cell degranulation upon co-incubation with target cells was determined by the expression of CD107a on the cell surface, which serves as a surrogate marker for NK cell degranulation [58]. In brief, overnight cultured NK cells enriched with the EasySep human NK cell enrichment kit (StemCell Technologies) from PBMCs from KIR2DL5A*001-positive donors or donors lacking KIR2DL5 genetically were co-cultured with CD155-expressing (CD155-transduced 721.221) or CD155-nonexpressing (721.221) target cells at an effector to target ratio of 1:2 in 200 μl complete R10 for 4 h at 37°C. During co-incubation, each well contained 2 μl anti-CD107a (clone LAMP-1, Biolegend) and 25μl/ml Brefeldin A. Cells were subsequently stained with LIVE/DEAD Fixable Near-IR and with the following antibodies: anti-CD3-BUV395 (clone UCHT1, BD), anti-CD16-PE-Cy7 (clone 3G8, Biolegend), anti-CD56-BV785 (clone NCAM16.1, BD), anti-KIR2DL5-PE (clone UP-R1, Biolegend) for 15 min at RT and fixed with CellFix (BD) before flow cytometric acquisition.

### Enrichment and stimulation of primary human CD4^+^ T cells

CD4^+^ T cells were isolated from fresh PBMCs trough negative selection with the EasySep human CD4 T cell enrichment kit (StemCell Technologies) according to the manufacturer`s protocol. After isolation, cells were cultured in complete R10 medium supplemented with 100U / ml IL-2 (Peprotech) and stimulated with anti-CD3/anti-CD28 Dynabeads (Life Technologies) for 3 days at 37°C / 5% CO_2_ at a bead to cell ratio of 1:2. Before infecting the stimulated cells, beads were washed out.

### Generation of HIV-1 virus stock from infectious molecular clones

HIV-1 viral stocks were produced as described previously [32]. In brief, plasmids harboring the full length proviral genome of infectious molecular clones of the primary strains CH077, CH164, CH185, CH198, CH236, CH293 (kindly provided by the Beatrice Hahn and John Kappes Laboratories) and the cell line-adapted strains NL4-3 and JR-CSF (National Institutes of Health (NIH); catalog no. 114 and 2708) as well as the respective *Δnef* mutants, which were generated previously [32], were used to transfect HEK293T cells. Therefore, 24 μg of DNA was diluted in Opti-MEM for a Lipofectamine 3000 transfection (Life Technologies) of HEK293T cells in a T75 flask according to manufacturer’s protocol. Samples were filled up to 10 ml with fresh cell culture medium (D10) and lentiviral particles were harvested 48 h after transfection. The supernatant was centrifuged at 500 x g, filtered through a 0.45 nm filter and concentrated by using Lenti-X concentrator (Clontech Labs). Viral stocks were aliquoted and stored at -80°C until further use.

### Infection of primary human CD4^+^ T cells with HIV-1 viral stocks

Stimulated CD4^+^ T cells were resuspended in the respective HIV-1 viral stock (Table 1) or in cell culture medium (mock control) and cells were spinfected for 2 h at 1.200 x g and 37°C. After spinfection, viral supernatant was removed and fresh complete medium (R10) supplemented with 100 U IL-2/ml was added to the cells. The infected cells were incubated for 72 h at 37°C/5% CO_2_ until antibody stainings for flow cytometry analysis took place. CD4^+^ T cells used for long-term co-culture with NK cell clones were infected for 4 h at 37°C without centrifugation to increase cell viability.

**Table 1:** HIV-1 strains used in this study.

Group	Virus	Subtype	Source (reference)
Cell line-adapted strains	NL4-3	B	Infectious molecular clone [59]
	JR-CSF	B	Infectious molecular clone
Primary strains	CH077	B	Infectious molecular clone (founder) [60]
	CH164	C	Infectious molecular clone (founder) [61]
	CH185	C	Infectious molecular clone (founder) [61]
	CH198	C	Infectious molecular clone (founder) [61]
	CH236	C	Infectious molecular clone (founder) [62]
	CH293	C	Infectious molecular clone (chronic) [61]

### Flow cytometry analysis of surface markers and intracellular staining of HIV-1-infected CD4^+^ T cells

To assess cell surface expression of proteins, flow cytometry was performed. Cells were washed with PBS and subsequently stained with the viability dye LIVE/DEAD Fixable Near-IR (Life Technologies) and with the antibodies anti-CD3-BUV395 (clone UCHT1, BD), anti-CD4-BV711 (clone RPA-T4, Biolegend), anti-CD155-PE (clone SKIL4, Biolegend), anti-HLA-ABC-Pe-Cy7 (clone W6/32, Biolegend), anti-HLA-E-BV421 (clone 3D12, Biolegend), anti-tetherin-APC (clone RS38E, Biolegend) and anti-IgG1-PE isotype control (clone MOPC21, Biolegend) for 15 min at RT. After washing the cells with PBS, an intracellular staining was performed. In brief, cells were incubated in BD Cytofix/Cytoperm for 20 min at 4°C, washed with BD Perm/Wash buffer and stained with anti-p24-FITC (clone KC57, Beckman Coulter) for 20 min. After another washing step, cells were fixed in BD Cellfix and analyzed by flow cytometry (BD LSR Fortessa). HIV-1-infected cells were defined as p24 Gag^+^ CD4^dim^ and uninfected as p24 Gag^-^ CD4^+^ cells. Cells infected with HIV-1 ΔNef mutant viruses were defined as p24 Gag^+^ and tetherin^-^ cell, as Nef-deficient viruses are not able to downregulate CD4.

### Generation of NK cell clones

NK cell clones were generated as described previously [63]. In brief, NK cells were enriched from PBMCs isolated from a KIR2DL5A*001-positive healthy donor and were subcloned by fluorescence activated cell sorting (FACS). Single NK cells, either expressing or not expressing KIR2DL5, were sorted into 96-well plates and cultured for 14 days in the presents of irradiated allogenic PBMCs and 8866 feeder cells at a ratio of 10:1 in NK cell cloning medium supplemented with 1 μg/ml phytohaemagglutinin (PHA (Invitrogen)). Cloning medium consisted of RPMI medium supplemented with 5% human serum (Sigma-Aldrich), 10% FBS (Sigma-Aldrich), 1X MEM-NEAA (Gibco), 1X sodium pyruvate (Gibco), 1X (2 mM) L-glutamine (Sigma-Aldrich), 100 μg/mL Primocin (Invivogen) and 200 U/mL IL-2 (Roche). After 14 days, outgrowing cells were transferred to a 48-well plate and cultured in NK cell cloning medium with frequent medium exchange and further expansion.

### NK cell co-cultivation with autologous HIV-1-infected CD4^+^ T cells

To analyze the antiviral potential of KIR2DL5^+^ NK cells, CD4^+^ T cells from the same donor from which NK cell clones were generated were stimulated and infected with HIV-1 CH198 or NL4-3, as described above. After 4 h of infection, CD4^+^ T cells were co-incubated with autologous KIR2DL5^+^ or KIR2DL5^-^ NK cell clones at an effector to target ratio of 2:1 in 300 μl complete R10 medium supplemented with 100 U/ml IL-2 (Peprotech) for 7 days. 100 μl of cell culture supernatant was replaced by fresh medium every 2 days. After 7 days, antibody staining with anti-CD3, anti-CD4, anti-tetherin, anti-CD16, anti-CD56 and intracellular staining with anti-p24 was performed and cells were analyzed by flow cytometry.

### Data analysis

Flow cytometry data were acquired on a BD LSR Fortessa (Biosciences) in the core facility

Flow Cytometry at the Leibniz Institute of Virology and analyzed using FlowJo software 10.7.1 (BD Biosciences). Data were statistically analyzed and graphically displayed in Graphpad Prism 9.0.1. Statistical analysis were performed using the non-parametric Mann-Whitney test or the Wilcoxon matched-pairs signed rank test for paired samples. If not indicated otherwise, mean values with standard deviations (SD) are shown for each group.

## Supporting information

S1 Fig KIR-Fc construct binding to CD155-coated beads. KIR-Fc construct binding to biotin, CD112 and CD155 measured by flow cytometry. Binding of KIR2DL1, KIR2DL4 or KIR3DL1 to biotin (neg. control), CD112 or CD155 was assessed as median fluorescence intensity (MFI) in three independent experiments (*n* = 3). The mean values of the experiments are shown as black bars and standard deviation is depicted as error bars.
(TIF)

S2 Fig Gating strategy to define CD69 expression on KIR Jurkat reporter cells. Gating strategy for flow cytometric analyses of CD69 expression on Jurkat reporter cells. Jurkat cells were first defined by forward scatter area (FSC-A) and side scatter area (SSC-A) characteristics. After doublet exclusion using forward scatter area (FSC-A) and forward scatter height (FSC-H), viable cells were identified as negative for LIVE/DEAD Near-IR staining (viability dye). Subsequently, gating on CD69 for the different conditions was performed (exemplary shown for co-incubation of Jurkat cells with biotin-, anti-KIR2DL5- or CD155-coated beads).
(TIF)

S3 Fig CD155 surface expression on 721.221 cells. Flow histogram shows expression levels of CD155 on the cell surface of parental 721.221 (CD155^-^) (white) and transduced 721.221 (CD155^+^) (gray) cells measured by using an anti-CD155 antibody.
(TIF)

S4 Fig HIV-1-mediated downregulation of CD155. CD155 expression levels were compared between HIV-1-infected (red) and uninfected (blue) CD4^+^ T cells. HIV-1-infected cells were determined by gating on p24^+^ CD4^dim^ cells and uninfected cells were defined as p24^-^ and CD4^+^. Histogram shows CD155 surface expression on HIV-1-infected (red), uninfected (blue) and mock-infected (black) CD4^+^ T cells, including isotype controls (HIV^+^ dashed black, HIV^-^ dashed grey).
(TIF)
