# Supplementary figures and images for: HIV-1 Nef-mediated downregulation of CD155 results in viral restriction by KIR2DL5+ NK cells
Source: PLoS Pathog. 2022 Jun 24;18(6):e1010572. doi: 10.1371/journal.ppat.1010572 (PMC9231786; doi:10.1371/journal.ppat.1010572)

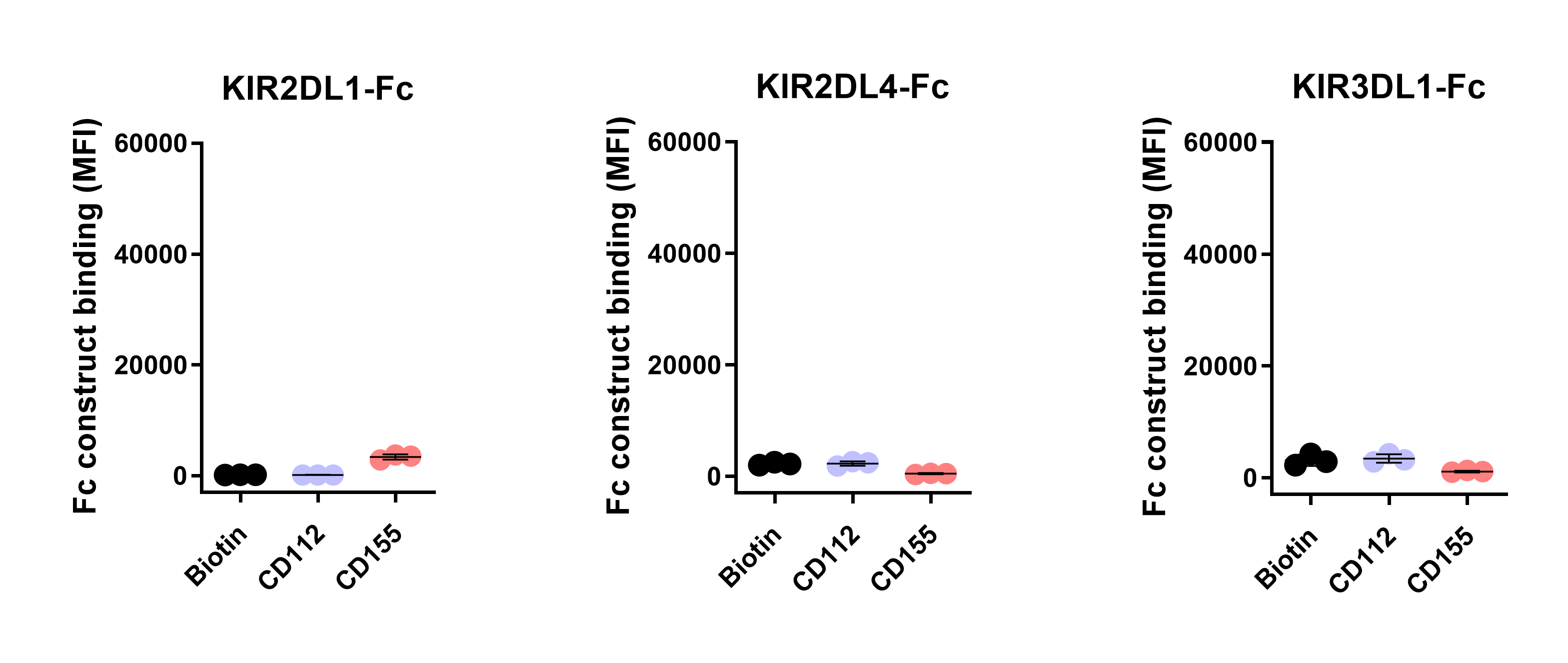

Supplement: S1 Fig — KIR-Fc construct binding to biotin, CD112 and CD155 measured by flow cytometry. Binding of KIR2DL1, KIR2DL4 or KIR3DL1 to biotin (neg. control), CD112 or CD155 was assessed as median fluorescence intensity (MFI) in three independent experiments (n = 3). The mean values of the experiments are shown as black bars and standard deviation is depicted as error bars. (TIF) [file ppat.1010572.s001.tif]

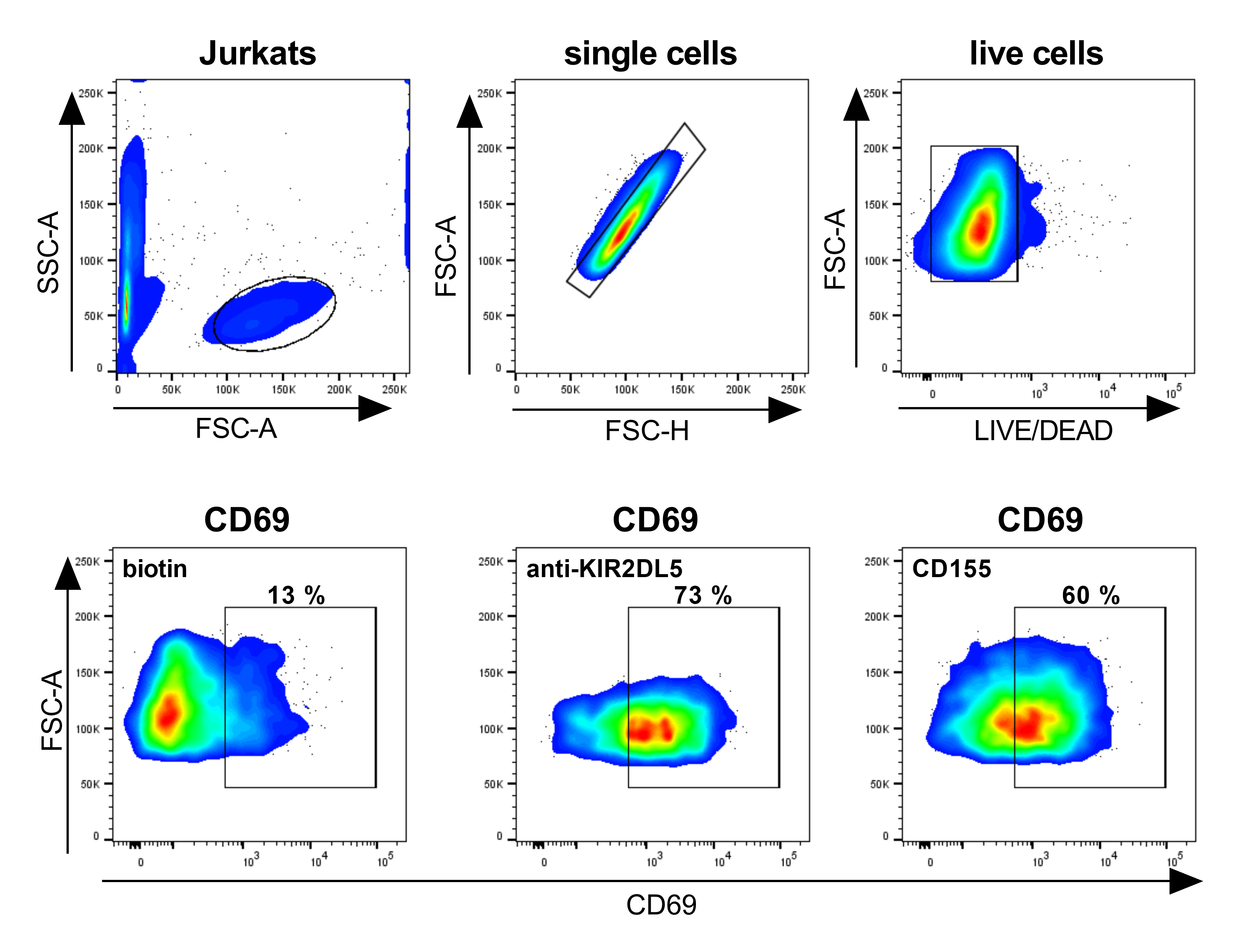

Supplement: S2 Fig — Gating strategy for flow cytometric analyses of CD69 expression on Jurkat reporter cells. Jurkat cells were first defined by forward scatter area (FSC-A) and side scatter area (SSC-A) characteristics. After doublet exclusion using forward scatter area (FSC-A) and forward scatter height (FSC-H), viable cells were identified as negative for LIVE/DEAD Near-IR staining (viability dye). Subsequently, gating on CD69 for the different conditions was performed (exemplary shown for co-incubation of Jurkat cells with biotin-, anti-KIR2DL5- or CD155-coated beads). (TIF) [file ppat.1010572.s002.tif]

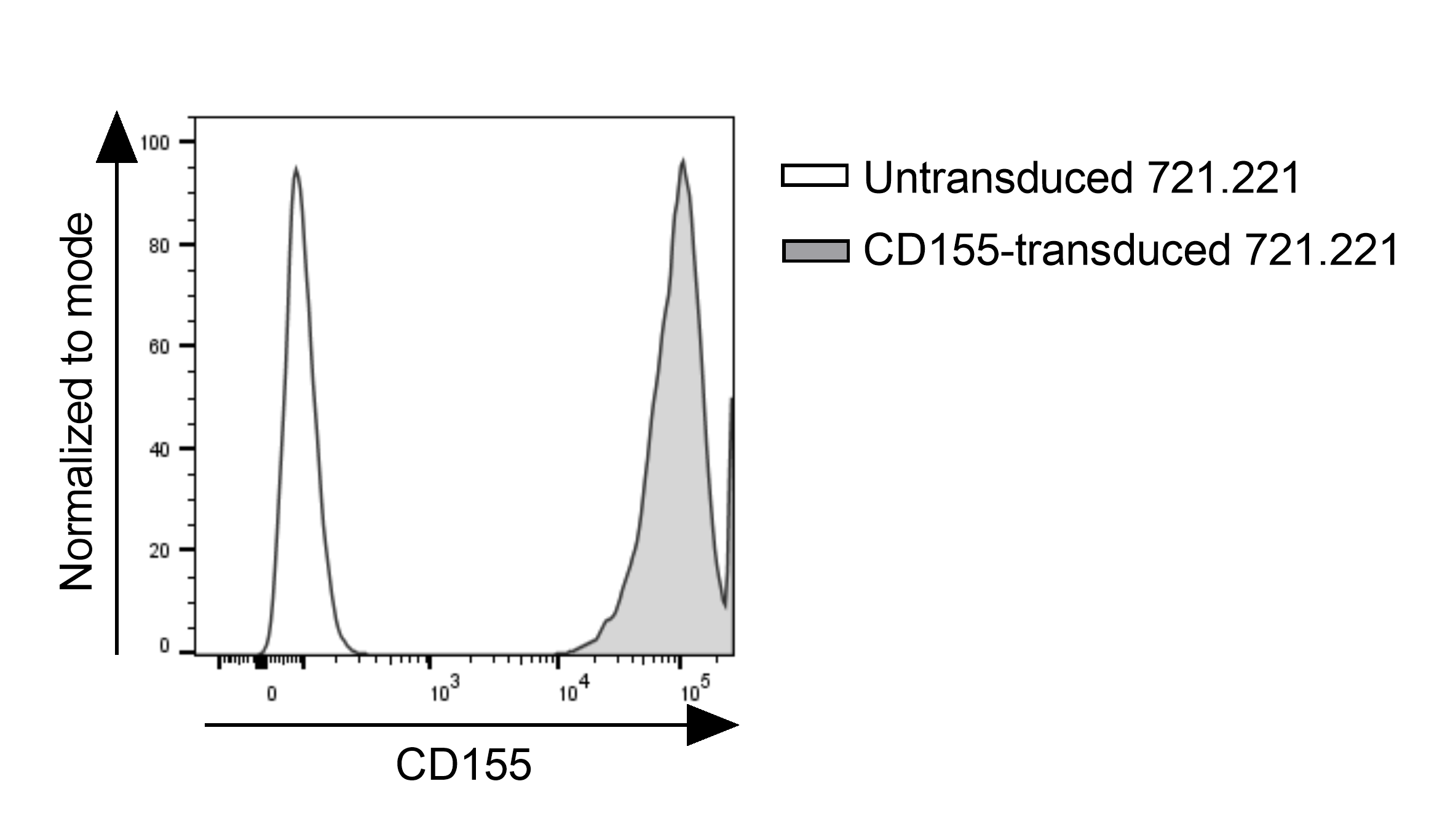

Supplement: S3 Fig — Flow histogram shows expression levels of CD155 on the cell surface of parental 721.221 (CD155-) (white) and transduced 721.221 (CD155+) (gray) cells measured by using an anti-CD155 antibody. (TIF) [file ppat.1010572.s003.tif]

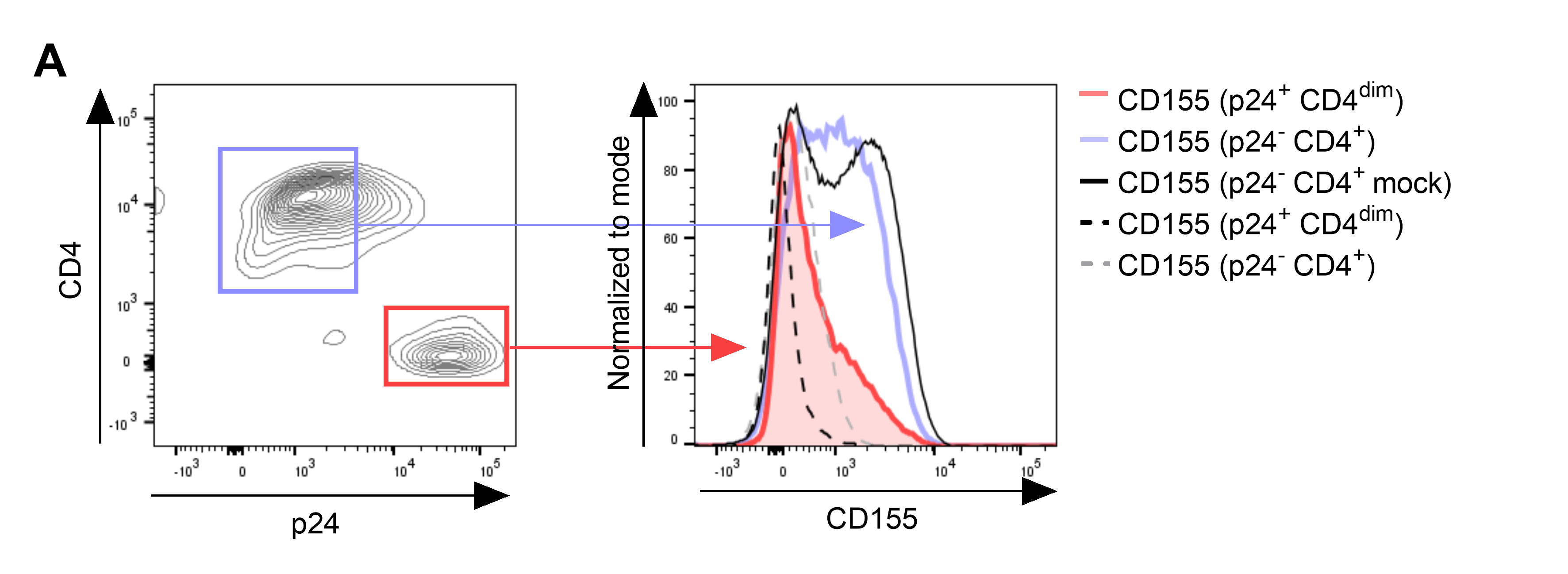

Supplement: S4 Fig — CD155 expression levels were compared between HIV-1-infected (red) and uninfected (blue) CD4+ T cells. HIV-1-infected cells were determined by gating on p24+ CD4dim cells and uninfected cells were defined as p24- and CD4+. Histogram shows CD155 surface expression on HIV-1-infected (red), uninfected (blue) and mock-infected (black) CD4+ T cells, including isotype controls (HIV+ dashed black, HIV- dashed grey). (TIF) [file ppat.1010572.s004.tif]
